# Optimizing the utilization of maternal and reproductive healthcare services among women in low-resourced Nigerian settings

**DOI:** 10.1186/s12889-023-16929-5

**Published:** 2023-10-23

**Authors:** Jacinta Chibuzor Ene, Henry Tochukwu Ajibo

**Affiliations:** Department of Social Work, University of Nigeria, Nsukka, Enugu State, 410001 Nigeria

**Keywords:** Healthcare, Maternal, Optimizing, Utilization, Reproductive, Women

## Abstract

**Introduction:**

Quality care delivery is an essential lifesaving interventions for maternal healthcare and reduction in mortality from preventable reproductive conditions. In African countries like Nigeria, numerous perceptions and militating factors present unique challenges in optimizing the utilization of maternal and reproductive healthcare services. As women continuously evolve away from the utilization of healthcare services, achieving universal health coverage for all emerges as a matter of concern.

**Method:**

A phenomenological and descriptive research design was used. The study participants comprised a total of 38 women selected from primary and tertiary healthcare institutions. They were purposively selected from four healthcare institutions in Nsukka, Enugu State, Nigeria.

**Result:**

Findings revealed that most rural women at the prenatal stage, utilize maternal healthcare services, but at the postnatal stage, they reject reproductive healthcare services owing to certain perceptions. Concerns about sub-optimal utilization of maternal and reproductive healthcare services were found under enabling, predisposing and need factors. Evidence-based interventions included instituting health insurance policies, improving the healthcare sector, personnel, collaboration among stakeholders, and grass-roots community education. Participants showed little knowledge of social workers’ engagement in healthcare institutions.

**Conclusion:**

Functional network of care between private and public healthcare system is the key to optimizing maternal and reproductive healthcare utilization. The study recommends stakeholder and community engagement in achieving functional networks of care, strengthening relational linkages between frontline health workers and equip rural women with better knowledge. All these are geared toward achieving optimal utilization of maternal and reproductive healthcare services among women in low-resourced Nigerian settings.

## Introduction

Across the globe, the contributions of women to the welfare of families, communities and nations are of utmost importance. As such, their health welfare during and after childbirth should be given great priority. This stems from the fact that a mother’s death has profound consequences on her family. Then, optimizing maternal and reproductive healthcare services utilization remains a mandatory step in accomplishing the Sustainable Development Goal (SDG):3 “to ensure healthy lives and promote well-being for all’ [34].

Globally, quality healthcare delivery is an essential lifesaving intervention. In most sub-Saharan African countries particularly Nigeria, the high burden of maternal and child mortality persists [26]. The country with 2.4% of the world’s population, contributes to 10% of global deaths for pregnant mothers [38]. Nigeria, further accounts for the fourth-highest maternal mortality rate of 576 per 100,000; with the second-highest child-birth death rate of approximately 262,000 babies [41]. It is important to note that above half of the maternal and neonatal deaths occur from attempted abortion, and pregnancy-related complications, particularly maternal hypertensive disorders, pre-eclampsia, hemorrhages, the premature rupture of membranes and infections [40]. As women age, they are vulnerable to illnesses including pelvic damage, cervical and breast cancer, fibroid, and other urogenital infections. Given the current challenges, it is envisaged that we will fall short of what is needed to realize the Universal Health Coverage (UHC) and SDG:3 target. This is a source of concern in the healthcare sector.

Maternal and reproductive healthcare services are delegated with providing adequate maternal and perinatal health, well-being and ending preventable feminine mortality [31]. It also encompasses monitoring of person-centered quality and respectful maternal and perinatal care, providing guidance, routine check-ups and recommending functional networks of care to optimize linkages for efficient and resilient healthcare systems [40]. Sadly, the feasibility of implementing a network of care to improve maternal and reproductive outcomes is sub-optimal [26]. The reason is that the quality of maternal care is a profound factor that impacts the delivery of a continuum of care among women in most of the sub-Saharan African countries. This situation subsequently increases women’s use of alternative healthcare services. For instance, it has been reported that 66% of livebirth categorized as non-institutional health delivery occurs in the presence of a mid-wife, relative or self-assisted birth [14]. Available evidence shows that in Nigeria, there is a deficiency in hygiene, contraceptive usage, and safe medication culture; which compromises patients’ safety [12, 19, 29]. Further, [28], posit that healthcare financing is the worst hit in the country, constituting 72% and 95% of public and private health expenditure in 2018 respectively.

Studies have shown that women’s ability to adopt routine check-ups and access appropriate treatment and services in health institutions is anchored on the various perceptions and factors of maternal and reproductive healthcare services utilization [27, 35, 42]. First is the perception of ante-natal and post-natal care services, delay in service delivery, fear of adverse health outcomes, cultural and religious assumptions [4, 11]. Again, factors including inequality in the distribution of health facilities between urban and rural areas, dilapidating equipment, inadequate number/attitude of health personnel, increasing corruption levels in the health sector, diseases, infections and high levels of household out-of-pocket payment for health goods and services, have been found in studies [15, 37]. Worrisome are concerns ranging from ignorance of women owing to their socio-demographic characteristics including age and educational status, occupation, influence of spouse decision; associated with low healthcare utilization and unhealthy living has been reported in the literature [14, 15].

Although in recent years, global investment in the health sector has been high. Nevertheless, the situation in the Nigerian healthcare system stems from reduced investment in the public health sector, high demand for healthcare services; a backlog of procedures, increasing cost of drugs, equipment and services [29]. Like other African countries, Nigeria operates a three-level healthcare sector including primary healthcare facilities, secondary health institutions (general hospitals) and a tertiary healthcare system (teaching hospital). These health institutions allocation is skewed; with a higher proportion going towards secondary and tertiary health institutions while the primary health facilities are neglected [24]. This is evident from the fact that negligence of the primary healthcare facilities results in women’s low utilization of maternal and health services particularly in the rural settings where it is predominant [21]. This falls short of the standard set by the World Health Organization’s initiative on safe motherhood.

Against these backdrops, it is needful to provide effective, quality, accessible and affordable healthcare services, particularly for rural women. This can be achieved through the engagement of stakeholders, communities and health workers in both the government and private sectors [26]. It is envisaged that efforts aimed at addressing this problem will lack some form of efficacy without the inclusion of medical social workers. This is incumbent on the roles social workers play in families, communities and collaboration with health practitioners. Specific to this context, social workers are expected to lobby, advocate, and intensify education on healthcare utilization [3]. They could push for a new narrative on early healthcare visitation, organize counseling and discourse sessions for support networks of patients, draw follow-up programs, enlighten and encourage them on compliance with health laws [25]. These efforts it is believed, will ensure an improved care system, optimal utilization of healthcare services and attaining a low proportion of maternal and neonatal child mortality. This is the gap this study hopes to fill.

In this study, therefore, an attempt is made to determine measures that will foster optimal utilization of maternal and reproductive healthcare services among women in low-resourced Nigerian settings. The study becomes important in Nsukka Local Government Area (L.G.A), against the background that there is a bias about rural areas in the implementation and monitoring of institutional healthcare facilities [13]. It is given the bias implicated in poor healthcare delivery, particularly in rural communities; that the study is anchored on the health service utilization model. Anderson and Newman’s [2] model of health service utilization serves as the theoretical underpinning of this paper. According to the model, the perception of maternal and reproductive healthcare service utilization by women is categorized under a series of factors including predisposing, enabling and need factor. The predisposing factors are socio-demographic characteristics including age, marital status, and parity (number of children), while the enabling factors include educational attainment, occupation and income [27]. In this study, however, the need factors adopted were the past experiences of rural women, service provider’s attitude and competencies, the cost of delivery, accessibility and environment of the institutional health facility. It then follows that the purpose of this study was to examine how these factors influence the perception and determine the utilization of maternal and reproductive healthcare services.

To date, numerous studies abound that have discussed optimizing maternal and reproductive healthcare services, particularly in sub-Saharan African countries, Nigeria inclusive [4, 15, 27, 42]. Of all these studies, none falls under the purview of medical social workers, particularly at the grass-roots level. This study, therefore, contributes to the existing literature on maternal and neonatal health, advocates for quality improvement in maternal and reproductive healthcare services, and ensures the integration of medical social workers in the rural community healthcare sector. It is against this background that this study poses the following research questions: (a) what perception holds on the utilization of maternal and reproductive healthcare services among women in low-resourced societies? (b) what factors influence the utilization of maternal and reproductive healthcare services among women in low-resourced societies? (c) what interventions can intensify the utilization of maternal and reproductive healthcare services among women in low-resourced societies? (d) what is the involvement of social workers in ensuring the utilization of maternal and reproductive healthcare services in Nigeria?

### Theoretical framework for optimizing maternal and reproductive healthcare services utilization

We anchor our theoretical framework on the model of health service utilization propounded by Anderson and Newman [2]. The model has been adopted by [5] to examine the use of health services by adolescent girls in South-East Nigeria. Further, [33] employed this model in investigating older adults’ behavior in receiving long-term services and support. The choice of this model is to understand and explain how and why people use certain types of health services. The model argues that the use of maternal and reproductive healthcare services by rural women is a function of their predispositions. This is categorized under need, enabling and predisposing factors. The key to the adoption of this model is to explore the determinants of women’s utilization of healthcare services, improve the quality of healthcare services and facilitate the policy-making process for equitable care and health services review.

In this study, we discovered that the predisposing factors (age and marital status) have a minor impact on health service utilization while the enabling (educational attainment, occupation and income) and the need factors (proximity of healthcare facilities, accessibility of health institutions, competencies and poor quality of care by health workers) are dominant predictors of maternal and reproductive healthcare utilization among rural women in Nsukka L.G.A. Further, women in this study aside various predispositions, expressed perceptions on cultural, religious and mystic assumptions as other determinants of maternal and reproductive healthcare utilization. It is against these backdrops, that we suggest the interventions of medical social workers in rural communities. We envisage that these professionals can help ensure optimal maternal and reproductive healthcare utilization among rural women by advocating for a functional network of care between the private and public care systems, adopting grass-root community health education, collaboration with health personnel, and indulging in home-health visits, among other measures. These suggestions, it is hoped, will optimize health services utilization, improve quality care program and reduce maternal and neonatal mortality in Nsukka, Enugu State, Nigeria.

## Methods

### Study design

In this current study, descriptive and phenomenological research designs with a qualitative research approach were employed in generating data. The descriptive research design aims to obtain in-depth information to systematically describe a phenomenon, situation or population [39]. Phenomenological approach is concerned with the perspectives and interpretations of people based on their experiences as it seeks to gain further insight into the thinking and behavior of women [10]. Employing these research designs, the researchers were able to describe in detail the narratives of women from low-resourced Nigerian settings on perceptions, challenging factors and measures to ensure optimal utilization of maternal and reproductive healthcare services.

### Study participants and recruitment

The study involved a purposive selection of three primary and one tertiary healthcare institution. The names of the health institutions are withheld for confidentiality and as requested by the health administrators. The study participants comprised a total of 38 women selected from four primary and tertiary healthcare institutions. The first category of participants were 27 expectant women in the Out-Patient Care (OPD) unit involved in three Focus Group Discussions. The second category consisted of nine women who have delivered and are awaiting discharge in the In-Patient Care (IPC) unit. Whereas, two medical social workers in a tertiary healthcare setting served as the third category of participants. The second and third categories of participants were involved in eleven in-depth interviews. It is important to note that equal participants were drawn from the three primary health institutions. Since there are no medical social workers in any of the primary healthcare institutions, the two social workers were drawn from one tertiary health institution.

### Study area

The participants for this study were drawn from Nsukka Local Government Area (LGA). Nsukka is situated in Enugu-North senatorial district within the southeast geo-political zone in Nigeria. The rationale for selecting the study area was because it operates a three level healthcare sector including the primary healthcare facility (Nsukka Primary Healthcare Center), secondary health institution (Nsukka General Hospital) and the tertiary system (University of Nigeria Medical Center) [16]gain, the locality is a semi-urban settlement with a major urban town, and 17 rural communities [36]. Hence, most rural indigenes owing to proximity, migrate to the town for healthcare services. In addition, studies have shown that the locality is one of the disease holo-endemic Nigerian communities with great economic burden on healthcare financing [22, 35]. The estimated population of Nsukka is 309,448 comprising 149,418 males and 160,030 females [8]. The locality has small-sized private (for-profit) health facilities but is dominated by government and mission health institutions [16, 23].

### Sampling procedure

For this study, the non-probability sampling method was employed. In this regard, purposive and availability sampling were deployed for selecting the study participants. The justification was that a unique target population was needed for the study. The target population comprised expectant women of up to six months, who are registered in the ante-natal care (OPC) service of the selected primary health facility. Also, women in post-natal care (IPC) who delivered at the time of this study and two medical social workers were selected. The study employed availability sampling to ensure that only available and willing participants were recruited in the study.

### Data collection

Data was collected using three focus group discussions and eleven in-depth interviews. Although in-depth interviews served as the main source of data collection, we were particularly interested in the rich data that is often generated in focus groups owing to the large number of ideas, issues and even solutions to a problem, as cited in qualitative studies [7, 9]. As social workers and researchers, we were also concerned with exploring and understanding the common and divergent experiences of rural women in the utilization of maternal and reproductive healthcare service in Nsukka, Nigeria, where medical social workers are not yet recognized. By understanding the common concerns and differing opinions, policies and programmatic actions can be initiated. Furthermore, the qualitative research method avail participants the opportunity to express their personal lived experiences with other women, while also providing them with the opportunity to learn from the tales of other women and reflect on the utilization of maternal and reproductive healthcare services. Informed consents to participate in the study were verbally obtained from the study participants after they were enlightened on the objectives of the study. The participants were assured of the confidentiality of every information or data they provided. They were also informed that they were at liberty to decline participation in the study at any stage of the interview if they no longer felt comfortable. This enabled the study participants to feel free and give out information without fear or prejudice. This informed the credibility and objectivity of the data obtained from the participants and the analysis process.

The researchers with two research assistances collectively developed the Focus Group Discussion (FGD) and In-Depth Interview (IDI) guides. They were made up of semi-structured questions and probes. According to [20], probes help in a deeper understanding of context through narratives that described the tales of women rather than a rudimentary guide. Three focus group discussions were held in the three primary health institutions. The data collection phase for the FGD started with some introductory activities. This allowed for the two research assistants who served as the note-taker and recorder to meet with the research participants informally so that they become more comfortable during the data collection process. Interviews and discussions took place between 5th February and 26th March 2023. Three Focus discussions and nine interviews were held in Igbo and English based on the convenience of the participants. These languages are familiar to the researchers, hence there was no need for an interpreter. In all, none of the interviews and discussions lasted more than 90 min to prevent fatigue.

In this study, we were guided by the phenomenological qualitative research approach which allowed us to discuss our participants’ experiences and concerns regarding our research topic [17]. This approach attaches importance to rich contextualized descriptions based on experiences and is free from pre-existing prejudices [32]. Again, it enabled us to adequately capture the participants’ phrases and nuances, see events as they appeared to them and as well minimize the impact of the researchers when collecting and analyzing data.

### Data analysis

The data analysis technique began with note-taking and careful audio-recording of all the discussions and interviews. Data were analyzed after transcribing the discussions and interviews in English language. This was to enable easy understanding. The researchers who did the transcriptions are grounded in Igbo language, though only a few participants expressed themselves in Igbo language. After translation and transcription, data immersion commenced by repeatedly hearing the audio and reading the transcribed discussion and field notes for familiarization. The data analysis process followed the qualitative procedure of data reduction, data display and conclusion. We compared the contents of the transcripts with the field notes and recorded information to ensure coherence.

Next, we coded the data in parent and child nodes. The coded data were further checked, grouped and categorized by other researchers. An inductive coding approach was adopted, meaning and themes were generated as we studied the transcript. This was done to ensure that codes with similar characteristics were grouped thematically to arrive at key themes. The use of thematic clusters to understand and communicate qualitative data is rooted in phenomenology [9]. Thematic analysis was employed to enable the researchers to categories the data into themes for easy comprehension. To increase the rigor of our analysis, two peers who did not participate in the study reviewed the dataset in keeping with peer debriefing and observer triangulation [30]. Their insights contributed to the final checks on the analysis. These exercises are in line with [10] overview of the qualitative research method. In this study, the final themes include:

- Perception of the utilization of maternal and reproductive healthcare services among women in low-resourced societies.
- Factors that influence the utilization of maternal and reproductive healthcare services among women in low-resourced societies.
- Interventions that can intensify optimal utilization of maternal and reproductive healthcare services among women in low-resourced societies.
- Assessment of the involvement of social workers in ensuring optimal utilization of maternal and reproductive healthcare services among women in low-resourced societies.

Finally, special connotations that addressed the research questions were pulled out as illustrative quotes from the thematic cluster to exemplify the key issues. Inscriptions were used to connote the study group (FDG- Focus Discussion Groups and IDI- In-depth Interviews).

### Ethical consideration

Ethical clearance for the study was sought from the institutional review board of the Strategic Contacts Ethics Publications (STRACEP) of the University of Nigeria, Nsukka campus, Enugu State; before the discussions and interviews commenced. All methods of data collected were carried out in accordance with relevant institutional guidelines and regulations. The need for written informed consent was waived by the institutional review board (IRB) of the Strategic Contacts Ethics Publications (STRACEP) of the University of Nigeria, Nsukka campus, Enugu State. The justification for the waived informed consent was because it was deemed unnecessary as the rural women volunteered to participate in the study. The participants were informed of their freedom to withdraw at any time in the course of the study. In all, they were assured of anonymity and confidentiality.

## Result

The results of our findings are presented in themes and subthemes. First, the researcher started by presenting the socio-demographic features of the study participants.

### Demographic characteristics of participants

In this study, there are 38 participants aged 30–59 years. A majority (80%) of the participants were younger women aged 30–44 years. They are predominantly Christians by religious affiliation and are married with children. Most (56%) attained and completed primary education, some (38%) attained but did not complete secondary and post-secondary school education, whereas few (16%) completed tertiary education. All the study participants are of the Igbo ethnic group. A good number were businesswomen and housewives while a few were civil servants. Most of the participants (67%) earned above 50,000 naira ($30) while others earned below.

#### Perception of the utilization of maternal and reproductive healthcare services among women in low-resourced societies

Concerning this view, the participants highlighted three key issues. First, some of them responded that the low utilization of maternal and reproductive healthcare services was due to cultural norms. Others were of the view that it remains a personal decision prompted by one’s religious belief and the preference for local birthplace owing to past experiences. A few participants stressed on the mystic assumptions associated with fear of knowledge of adverse health outcomes. Listed below are some typical quotes:

Traditionally, childbearing is used to assess a woman’s strength and actual age. Under normal circumstances, women wish to adopt normal childbearing methods. Oftentimes, complications may occur and there may be a need for alternative (cesarean operation) childbearing methods. Expectant women may decline from the utilization of maternal health facilities so that they can have normal childbearing **[FGD, Trader, 36 years].**

Actually, my religion is against the use of reproductive healthcare services. For instance, the insertion of birth control items is against my faith. But, in the facilities, it is recommended to enable the spacing of children. I only use the natural method **[IDI, Housewife, 43 years].**

Aside, the ante-natal and post-natal healthcare, I do not need maternal healthcare services. The reason is that I can only attend medical examinations when I am sick. But when you visit maternal health institutions for medical examination; certainly you may be informed of one sickness that has emerged and this is very scary **[IDI, Trader, 31 years].**

Years back, when I went for a medical examination, I was informed of having kidney stone. I rejected this information. It was later found that I had kidney inflammation, which required the consumption of water. The false medical information almost affected my wellbeing **[FGD, Civil servant, 42 years].**

Often, people have the perception that unknown sickness does not kill, but what kills is known ill-health. This is because you are only afraid of what you know and so it is better that you do not know of any ill-health you have till it evolves. After all, we must die someday **[FGD, Civil servant, 39 years].**

Indeed, I attend ante-natal and post-natal child care services before and after birth delivery. But I do not give birth in healthcare facilities because I choose to give birth in local birth homes. The mid-wives talk gently and will encourage you through the painful delivery period. This help to reduce the pains in childbearing **[IDI, Housewife, 30 years].**

Thus, from the discussions, we found that women’s perception of the utilization of maternal and reproductive healthcare services is influenced by cultural norms, religious beliefs, past experiences and mystic assumptions. Based on these impediments, they belief that utilization of reproductive healthcare services anchors on fear of adverse health outcomes. Few participants, however, felt that utilization of maternal and reproductive health services was a determinant of choice and not a necessity.

#### Factors that influence the utilization of maternal and reproductive healthcare services among women in low-resourced societies

In this theme, we present the factors that influence the utilization of maternal and reproductive healthcare services among women. Contrasting views were raised by the participants in this regard. While some participants noted the enabling factors including women’s educational status, occupation and economic level, others reported need factors like proximity of healthcare facilities, accessibility of health institutions, and poor quality of care by health workers. Few participants stressed on predisposing factors like age and marital status of women.

#### Enabling factors

Though some participants deemed it necessary for women to utilize healthcare facilities during and after maternal care, others had disregard due to factors such as women’s educational status, occupation and economic level. They emphasized that women are most affected by these factors owing to gender marginalization which prevails in the study locality. Hear these quotes:

Women are stereotyped in our society because we are in a patrilineal environment. Our education is assumed to be irrelevant, so they are denied equal educational opportunity with the males. Our low educational status affects our occupation and level of income. This discourages healthcare utilization because educated women are very much aware of the importance of routine reproductive health check-ups and maternal care in health facilities **[FGD, Civil servant, 47 years].**

The cost of service charges in health facilities is expensive. For instance, I have observed that the use of non-institutional health facilities is because of service charges. In most cases, the cost of service charged often results in premature discharge. This also patterns to other healthcare services where you pay for routine check-ups, contraceptives and other services **[IDI, Civil servant, 34 years].**

#### Need factors

There was a consensus on the need factors that influence the utilization of maternal and reproductive healthcare services among women in low-resourced societies. The factors highlighted by the participants include proximity of healthcare facilities, accessibility of health institutions, competencies and poor quality of care by health workers. Below are the responses from the participants:

Our government is ignorant of what is happening to us. The hospitals are far from us and the health centers are not well equipped. We just have to use what we have to help ourselves. We cannot die going to those far places or go to the health center in our community where you will not see anybody at night when you want to give birth. Why will I go to a government hospital when there is a good birth center in our community that is doing the same thing and even better than all these private and government hospitals **[FGD, Businesswoman, 40 years]**.

In my last pregnancy which began at midnight, my husband took me to the hospital where I registered. I was in the waiting room for over 20 min before one of the health workers reluctantly went to pick up my folder from the shelf. The other healthcare provider was in deep sleep. By the time the other who went to get my folder came back, I have already delivered my baby in the waiting room **[FGD, Trader, 32 years]**.

While this participant was still sharing her personal experience, another participant quickly added:

When you are in labor pains, some healthcare providers use abusive language. Some also inform the woman in labor not to disturb them with her screams as they were never consulted during her sexual relationship **[FGD, Housewife, 28 years].**

Many of the study participants though acknowledged the fact that healthcare assistance is very stressful, they were however of the opinion that healthcare provision especially with expectant mothers should be carried out with tenderness and care. Most of the study participants identified challenges such as absenteeism, negligence, long waiting time, health provider competencies, and unfriendly attitude among others; as the reasons that discourage the utilization of maternal and reproductive healthcare services among women in low-resourced societies.

#### Predisposing factors

Narratives from the participants show that age is a strong predisposing factor. For instance, Some IDI participants replied, “*some young women are very fast on issues regarding reproductive health. They give birth in their homes and use birth control measures such that they do not have unwanted pregnancies*”. Yet, another participant explained, “*most older women are naive, ignorant and unconcerned about reproductive healthcare. They feel that they have passed the reproductive stage*”. In the course of the discussions, other participants stressed on the marital status of women. For instance, hear this participant:

Marital status cannot influence the utilization of maternal and reproductive healthcare. This is because most men particularly those in the rural areas only use their wives for entertainment. It is the woman who bears all the pain and will decide to adopt appropriate maternal and reproductive healthcare services **[IDI, Housewife, 37 years].**

For another IDI participant, she reported: *“whether you adopt reproductive healthcare services or not, the husband’s consent must be sought and prevails*”. She narrated a sad story of a friend who after two months of the fifth child delivery, adopted birth control measures. When the husband realized her decision, it resulted in a severe fight that claimed the woman’s right eye.

In this regard, we see that there were contrasting views regarding the influence of male spouses in the utilization of maternal and reproductive health services. While some participants were of the view that the male spouses have no influence on maternal and reproductive healthcare utilization, others disagreed.

#### Interventions that can intensify optimal utilization of maternal and reproductive healthcare services among women in low-resourced societies

The participants gave various suggestions to promote the utilization of maternal and reproductive healthcare services in low-resourced societies. They mentioned interventions including implementation of healthcare policies, improved healthcare facilities and personnel through capacity building, equity in the health sector categories, mobile health services, community-based intervention and education for low-resourced dwellers. Interventions highlighted were categorized under two sub-themes including government support and community-based interventions.

#### Government supported interventions

Some participants suggested that they want government legislators to implement health policies and laws that will ensure free healthcare for all. They emphasized that this is greatly needed particularly for the vulnerable women at the grass-root. For a participant, she stressed that the implementation of health laws will ensure regular utilization of health institutions. This will encourage and increase their knowledge of available healthcare services. Additionally, some participants explained that periodic routine check-up exercises should be conducted in rural communities to encourage regular healthcare utilization. This can be done through a collaborative effort of local leaders and health workers. See a unique quote below:

Government should institute mandatory health laws and policies. This should be enforced with collaboration from local chiefs, religious leaders, health workers and security officials. With their effort, women can gain access to basic reproductive health services, while knowledge of disease-causing infections and illness can be reduced **[IDI, Civil servant, 47 years].**

Another suggestion raised by some participants was on improved healthcare facilities, equity in the distribution of funds among the three health sector categories and ensuring personnel capacity building and training on healthcare. Regarding these views, the majority of the FGD participants were quick to point this out. For an IDI participant, she responded: “*knowledge can be transferred*. *It is simply based on what you have acquired that you can transfer to another person”.* Yet another said; “*the world is ruled by medical technology. It is then essential that the primary healthcare facilities should be improved like other sectors to ensure maximum service delivery*”. Other illustrative quotes include:

Health workers must indulge in regular training. They could go for online courses, vacation study programs and the like. This will help increase their knowledge on treading health technologies and evolving diseases **[IDI, Social worker, 47 years].**

Seminars could be organized periodically for health workers. This would keep them informed on current and vital health issues. This also calls for increased health workers, especially in rural sectors **[IDI, Housewife, 37 years].**

#### Community-based intervention

Lastly, some participants gave suggestions on community-based intervention through education and enlightenment. This becomes necessary based on the fact that some individuals had doubts concerning the effectiveness of certain medical technologies. Others reported that aside from effective medical technologies, some reproductive diseases can be prevented. This can be done through adherence to health guidelines and safe medication. Below are illustrative quotes:

In this locality, some women have refused to accept health technologies like contraceptives that will ensure adequate wellbeing. They belief that their forefathers received none of these contraceptives but they lived long. So, adopting medical technologies and drugs will only reduce their body’s immunity. Hence, when they need to have children, reproduction may not occur **[IDI, Trader, 32 years]**.

The ignorance of some individuals has led to certain diseases still in existence. Community education and enlightenment are greatly needed. This can be done with the assistance of local Chiefs, women leaders and health workers. Vital health knowledge should be made available to everyone despite the gender. This will help in dispersing health awareness to others **[IDI, Social worker, 41 years].**

#### Assessment of the involvement of social workers in optimizing the utilization of maternal and reproductive healthcare services

In this regard, the resounding view was that there is little or no knowledge about social work practitioners’ involvement in healthcare settings. To use evidence in informing the involvement of social workers in this context, we gleaned responses from the participants to understand connecting narratives. For instance, a participant responded “*I do not know them and who they are*”. Yet another reported, “*I have heard of one in this hospital, but I do not know their roles here”.*

Stressing on this view, the two interviewed social workers explained that formal social work practice in Nigeria is relatively new and emerging. This implies that it is still unpopular, especially in low-resource community where the study was conducted. Observe these illustrative quotes:

In the healthcare settings, medical social work practitioners collaborate with health personnel. In this country, the professionals face an uncertain future. Medical social workers are being pushed to rethink their mission and identify the practice components and gaps needing their expertise. **[IDI, Social worker].**

The roles of social workers in the health setting are numerous. They educate patients and families, provide care assistance, indulge in-home visits and follow up in special cases. Their services are essential for quality healthcare delivery **[IDI, Social worker].**

## Discussions

Despite the growing availability of medical technology, modernization and government effort in the healthcare sector, achieving optimal healthcare service utilization remains a common problem in low and middle-income countries, Nigeria inclusive. With an increasing population in the country [38] and to achieve the World Health Organization [40] initiative on safe motherhood, optimizing the utilization of healthcare services becomes paramount. Findings show that rural women in this study abscond from utilizing institutional health facilities owing to three major perceptions. First, was the perception of cultural norms that assess a woman’s strength and age based on the normal child delivery methods as against the alternative method (cesarean operation). Second was that their religious beliefs are against the utilization of reproductive healthcare services. Third was the mystic assumption of fear of adverse health outcomes. These perceptions are anchored on their decision to utilize health facilities as a determinant of choice and not a necessity. This finding is a clear indication that optimal health facility utilization must be advocated as evidenced by several studies [12, 18]. This will ensure early identification of ill-health, adequate treatment, and reduced health migration; to prevent the rapid spread of illnesses and increasing mortality.

Quite disturbing is the finding that gender marginalization is a determinant in optimizing maternal and reproductive healthcare services. This still prevails in most African countries like Nigeria. It was found that gender marginalization in the study locality affects women’s educational status, occupation and economic level. In this regard, the education of females is restrained resulting in inadequate investment by families on female education. This discriminatory practice interacts closely with poverty and consequently, hinders the opportunity of obtaining good jobs and substantial income. It is important to note that in Nigeria like some African countries, the traditional society allows inequitable gender norms which prioritize feminine roles as wives, mothers, and household caretakers [22]. Alternatively, the education of the girl-child is restrained to informal domestic education at home, on hygiene, cooking, laundry and general home management. Oftentimes, knowledge gained from the informal setting increases their sub-optimal utilization of health facilities. To fill the gap in maternal health, there is a need to advocate for community-based health education and enlightenment programs, particularly on regular health facility utilization as suggested by [26]. This can be done during ante-natal care services which record an increasing number of women in attendance according to studies by [6, 31]. This finding corroborates with existing literatures by [1] and [20] on the essence of health education during ante-natal care as a measure in achieving maternal healthcare service utilization for rural women in Nigeria.

A novel finding from this study is that the cost of service charged in healthcare institutions poses as the most challenging enabling factor. This is sequentially and logically true given that women are economically vulnerable [6]. With their low economic status, they are more likely to disregard health service utilization, particularly in the choice of birth location, contraceptives and routine reproductive check-ups. For example, routine reproductive health check-ups for cancer of the breast, cervical, obstetric complications and other injuries on the reproductive organs require adequate screening. These health issues when identified require effective counselling and treatment. Consequently, rural women with lower income will not afford the service charged for screening and treatment of these preventable health issues. Evidence from arrays of literature corroborate with the foregoing result on the under-utilization of healthcare services owing to low income in Nigeria [28, 35, 37]. We then recommend healthcare policies implementation, equity in funding of the three categories of health sectors and exploration of health innovations. All these could help resolve the out-of-pocket health expenditure and non-utilization of healthcare facilities by women in Nigeria.

Indeed, providing medical assistance can be demanding, overwhelming and challenging [14]. It is against this background that we argue that strategizing quality maternal care for optimal health utilization is essential in the Nigerian healthcare system. We observed that another major challenge that hindered maternal and reproductive health service utilization was the accessibility of health institutions. However, other need factors identified were health providers’ incompetence, absenteeism, and negligence; all anchored on the poor quality of care by health workers. We see in a developed context, that functional networks of care can strengthen relational linkages between frontline health workers and provide evidence-based maternal and reproductive care and services. It can also provide guidance and recommendations on models and networks of care, re-visioning the attaining of Global Strategy for Women’s, Children and Adolescents’ Health (WHA69.2), and strengthen private sector engagement for Universal Health Coverage, especially in low-and middle-income settings [41].

Our findings demonstrated that since health is an uncompromising issue, then interventions for optimal utilization of healthcare facilities become essential. Corroborating other studies that advocated for improving the Nigerian healthcare sector [24, 31], we observed various suggestions that pointed to government-supported intervention. First, they suggested the implementation of health laws like health insurance policies, for all citizens as obtained in developed countries. Second, they reported on improved healthcare facilities by ensuring equity in the primary, secondary and tertiary health institutions. Third, participants stressed on personnel capacity building and training on quality healthcare service delivery. These strategies they highlighted will generate programmatic-focused evidence, develop guidelines, norms, standards, and support regions in adaptation, implementation and monitoring of person-centered quality and respectful maternal and reproductive care. In all, they point to the pivotal obligation of the government and all health workers.

Another unique finding in this study that is not common in other studies is the involvement of social workers in healthcare institutions. Quite disturbing is the finding that the participants did not know of social workers’ involvement in the health care setting. This could be related to the fact that formal social work practice is relatively new and emerging in Nigeria [25]. These professionals with their skills in community mobilization, can advocate and intensify health education on early and regular health facility visits. They could equally organize health programs including mobile healthcare, voluntary care services, and follow-up home visits among other healthcare services [3]. More so, social workers can collaborate with medical professionals and caregivers in providing quality care networking for patients, particularly at the grass-root. Finally, social workers are key to galvanizing discourse sections for support networks of family members (male spouses), through advocacy, partnerships, policy dialogue and capacity building [12]. This will fill the gap in the unavailable but needed health personnel, improve the functional care system and ensure optimal utilization of maternal and reproductive healthcare services among women in low-resourced Nigerian settings.

### Strength and limitations

The researchers first encountered difficulties in recruiting respondents and participants who would volunteer for the study. Second, the participants were all women, selected from a particular locality. These limitations notwithstanding, we believe that the findings of this study remain valid.

## Conclusion

Equity in healthcare remains a core concern in attaining universal health coverage. Rural women constitute a vulnerable group whose health needs should be considered with utmost importance. This study then reveals that:

First, to ensure healthy living and promote wellbeing for all women, healthcare service utilization becomes paramount. This is pegged on advocating for regular utilization of health facilities despite the cultural norms and perception. This effort will ensure early identification of health status and adequate treatment to prevent rapid spread of the ill-health and increasing mortality.

Second, urgent attention is needed to eliminate gender marginalization. Sadly, this norm prevails in most African countries. Eliminating this discriminatory practice will not only increase the utilization of maternal and reproductive health service but will encourage investment by families in female education and the opportunity to obtain good jobs and substantial income.

Third, owing to our cultural norms, women are often economically vulnerable. This challenge consequently hinders healthcare service utilization. In this regard, healthcare policy implementation and mainstreaming the primary health sector (where the bulk of rural women obtain health services) into the national health insurance scheme will help in achieving the universal health coverage and the Sustainable Development Goal (SDG):3.

Lastly, healthcare assistance could also emanate from the inclusion of medical social workers in health institutions. The collaboration of social workers with health professionals becomes essential with the aim of providing quality care networking for patients, particularly women at the grass-roots.

## Data Availability

Data are stored in coded materials and databases without personal data, and the authors have policies in place to manage and keep data secure. All datasets generated and/or analyzed during the current study are not publicly available due to the principle of confidentiality assured to the participants and mode of storage (tape recording device), but are available from the corresponding author on reasonable request.
